# Observational study of lenalidomide in patients with mantle cell lymphoma who relapsed/progressed after or were refractory/intolerant to ibrutinib (MCL-004)

**DOI:** 10.1186/s13045-017-0537-5

**Published:** 2017-11-02

**Authors:** Michael Wang, Stephen J. Schuster, Tycel Phillips, Izidore S. Lossos, Andre Goy, Simon Rule, Mehdi Hamadani, Nilanjan Ghosh, Craig B. Reeder, Evelyn Barnett, Marie-Laure Casadebaig Bravo, Peter Martin

**Affiliations:** 1 Department of Lymphoma/Myeloma, University of Texas MD Anderson Cancer Center, 1515 Holcombe Boulevard, Houston, TX USA; 2 Abramson Cancer Center of the University of Pennsylvania, Philadelphia, PA USA; 3 University of Michigan, Ann Arbor, MI USA; 4 Sylvester Comprehensive Cancer Center, Division of Hematology Oncology, University of Miami, Miami, FL USA; 5 John Theurer Cancer Center at HUMC, Hackensack, NJ USA; 6 Department of Haematology, Derriford Hospital and Plymouth University Medical School, Plymouth, UK; 7 Medical College of Wisconsin & CIBMTR, Milwaukee, WI USA; 8 Carolinas HealthCare System, Levine Cancer Institute, Charlotte, NC USA; 9 Mayo Clinic Scottsdale/Phoenix, Scottsdale, AZ USA; 10 Celgene Corporation, Summit, NJ USA; 11 Celgene International Sàrl, Boudry, Switzerland; 12 Weill Cornell Medical College, New York, NY USA

**Keywords:** Ibrutinib failure, Lenalidomide, Mantle cell lymphoma

## Abstract

**Background:**

The observational MCL-004 study evaluated outcomes in patients with relapsed/refractory mantle cell lymphoma who received lenalidomide-based therapy after ibrutinib failure or intolerance.

**Methods:**

The primary endpoint was investigator-assessed overall response rate based on the 2007 International Working Group criteria.

**Results:**

Of 58 enrolled patients (median age, 71 years; range, 50–89), 13 received lenalidomide monotherapy, 11 lenalidomide plus rituximab, and 34 lenalidomide plus other treatment. Most patients (88%) had received ≥ 3 prior therapies (median 4; range, 1–13). Median time from last dose of ibrutinib to the start of lenalidomide was 1.3 weeks (range, 0.1–21.7); 45% of patients had partial responses or better to prior ibrutinib. Primary reasons for ibrutinib discontinuation were lack of efficacy (88%) and ibrutinib toxicity (9%). After a median of two cycles (range, 0–11) of lenalidomide-based treatment, 17 patients responded (8 complete responses, 9 partial responses), for a 29% overall response rate (95% confidence interval, 18–43%) and a median duration of response of 20 weeks (95% confidence interval, 2.9 to not available). Overall response rate to lenalidomide-based therapy was similar for patients with relapsed/progressive disease after previous response to ibrutinib (i.e., ≥PR) versus ibrutinib-refractory (i.e., ≤SD) patients (30 versus 32%, respectively). The most common all-grade treatment-emergent adverse events after lenalidomide-containing therapy (*n* = 58) were fatigue (38%) and cough, dizziness, dyspnea, nausea, and peripheral edema (19% each). At data cutoff, 28 patients have died, primarily due to mantle cell lymphoma.

**Conclusion:**

Lenalidomide-based treatment showed clinical activity, with no unexpected toxicities, in patients with relapsed/refractory mantle cell lymphoma who previously failed ibrutinib therapy.

**Trial registration:**

Clinicaltrials.gov identifier NCT02341781. Date of registration: January 14, 2015

**Electronic supplementary material:**

The online version of this article (10.1186/s13045-017-0537-5) contains supplementary material, which is available to authorized users.

## Background

Mantle cell lymphoma (MCL) accounts for 3 to 6% of non-Hodgkin lymphomas and is generally characterized by cyclin D1 overexpression and, more recently, by SOX11 expression [1–3]. MCL is generally considered incurable with standard chemoimmunotherapy and approved targeted agents [4]. Although multiple molecular-based therapies have improved outcomes for patients with relapsed/refractory MCL, there is no established standard-of-care [5, 6]. As summarized in a recent review, various chemoimmunotherapy regimens tested in small clinical trials in this setting have achieved high overall response rates (ORR) ranging from 58 to 93%, but progression-free survival (PFS) has been limited to < 2 years [6], with reported overall survival (OS) as < 3 years [7–9].

Bortezomib, lenalidomide, and ibrutinib have received US Food and Drug Administration (FDA) approval for the treatment of relapsed/refractory MCL [10–12], and lenalidomide, ibrutinib, and temsirolimus are registered for this indication in the European Union [10, 13, 14]. Monotherapy activities with these targeted agents in phase II studies report ORRs ranging from 22 to 68%, complete response (CR) rates ranging from 2 to 21%, and median duration of response (DOR) ranging from 9.2 to 19.6 months [6]. In a randomized study comparing two targeted agents in patients with relapsed/refractory MCL, ibrutinib significantly reduced the risk of progressive disease (PD) or death compared with temsirolimus (hazard ratio [HR] 0.43; 95% confidence interval [CI], 0.32–0.58; *P* < 0.0001) [15]. After a median follow-up of 20 months, ibrutinib demonstrated an improved median PFS (14.6 versus 6.2 months; *P* < 0.0001), 2-year PFS (41 versus 7%; *P* value not reported), ORR (72 versus 40%; *P* < 0.0001), and CR rate (19 versus 1%; *P* value not reported) compared with temsirolimus.

Although these treatments have shown significant antitumor activity and are commonly used, primary and acquired resistance, intolerance, and drug-related toxicities are significant limitations of these treatment approaches. With ibrutinib in particular, recent studies have shown that MCL patients with primary or acquired resistance have poor clinical outcomes. A retrospective review of 31 patients with MCL who had PD following discontinuation of ibrutinib and received salvage chemoimmunotherapy showed an ORR of 32% with the first salvage regimen and an estimated 22% 1-year OS (median 8.4 months) [16]. In another retrospective analysis, 114 heavily pretreated patients with MCL developed PD, while on ibrutinib (for a median treatment duration of 4.7 months) had a median OS of 2.9 months after discontinuing ibrutinib [17].

The oral immunomodulatory drug (IMiD®) lenalidomide has demonstrated antitumor activity in preclinical studies of MCL, both as monotherapy and in combination with rituximab [18–21]. In clinical trials in patients with relapsed/refractory MCL and other non-Hodgkin lymphomas, lenalidomide demonstrated activity when used as a monotherapy [22–28] and in combination with rituximab (R^2^) [29, 30].

The objective of this retrospective, observational, multicenter MCL-004 study (NCT02341781) was to evaluate the clinical effectiveness and safety of lenalidomide used as monotherapy and in combination regimens to treat patients with MCL who had relapsed/progressed to an ibrutinib-containing treatment (i.e., had an initial response of PR or better) or who were refractory to (i.e., best response of SD or worse) or unable to tolerate ibrutinib.

## Methods

### Patients

Harmonization E6 requirements (Good Clinical Practice) and ethical principles per the Declaration of Helsinki were followed. All aspects of the study were reviewed with the study investigators and staff; accuracy was confirmed through source data verification.

Inclusion criteria were age ≥ 18 years; MCL verified by investigator review of a pathology report; at least one dose (cycle 1, day 1) of ibrutinib (monotherapy or combination); and ibrutinib failure defined as relapse (CR followed by relapse at any time), PD (PR followed by PD at any time), refractory (PD, or stable disease [SD] followed by PD, while on ibrutinib), and/or intolerance (discontinuation of ibrutinib for reasons other than PD). Lenalidomide was not required to immediately follow ibrutinib.

### Study design

After identifying MCL patients treated with or intending to take lenalidomide following ibrutinib failure, an informed consent document was completed by the patient (family member/legal representative if patient was deceased), or a waiver was granted from the Institutional Review Board or Ethics Committee (IRB/EC) if consent was deemed not necessary for data collection. Patients were then enrolled into the clinical database, and data were extracted from medical charts including demographic information, relevant medical history, baseline disease characteristics, date of initial MCL diagnosis with pathology report, prior therapies (including treatment dates and best response), ibrutinib and lenalidomide treatment dates and outcome, copy of imaging reports, date of last follow-up/disease status, documentation of adverse events (AEs), and date/cause of death. Patients were enrolled after meeting eligibility criteria. Non-retrospective data may have been collected when lenalidomide was ongoing at study entry.

The primary endpoint was ORR defined as achievement of CR or PR per 2007 International Working Group (IWG) response criteria [31]. When initial assessments used IWG 1999 criteria (i.e., unconfirmed CR [32]), the corresponding response per IWG 2007 was changed to PR. Patients without a response evaluation or had an unknown response were considered non-responders. The secondary endpoint was DOR (time from initial response to lenalidomide-based therapy of ≥PR to relapse/PD/death, whichever occurred first). Responding patients without PD/death at analysis were censored at the last assessment date.

### Response and safety assessments

Time-to-event data were estimated using the Kaplan-Meier method [33]. Planned analyses were conducted for MCL subgroups of refractory (best response to ibrutinib of SD or worse), relapsed/PD (initial response to ibrutinib of ≥PR followed by PD), and those unable to tolerate ibrutinib (any reason other than lack of efficacy).

Available records of treatment-emergent AEs (TEAEs) with an onset date after lenalidomide initiation through 28 days after the last lenalidomide dose, regardless of causality, were analyzed in the safety population. AEs were classified according to the National Cancer Institute (NCI) Common Terminology Criteria for Adverse Events (CTCAE) version 4.03.

### Statistical analysis

All efficacy evaluations were conducted in the eligible patients. Patients were grouped by first type of lenalidomide treatment received: single agent, in combination with rituximab, or in combination with other agents. The response rate probability was estimated using the proportion of responding patients with an exact two-sided 95% CI; a sample size of 30 patients would allow a two-sided 95% CI (lower boundary of 10%) for an expected proportion of 25%.

## Results

### Patient characteristics

MCL patients from March 1, 2009, to April 12, 2016 who were treated with lenalidomide following ibrutinib therapy were enrolled. The data cutoff for all patients was November 1, 2016. The study enrolled 58 patients at a total of 11 study sites, including 10 sites in the USA and one site in England (Additional file 1: Table S1). Seven patients signed informed consent forms (one patient signed consent prior to initiating lenalidomide treatment), and 51 patients had IRB/EC waivers. Thirteen patients were treated with lenalidomide monotherapy, 11 with lenalidomide plus rituximab, and 34 with other lenalidomide combinations (Additional file 1: Table S2). Two patients initially identified for analysis were excluded from this observational cohort because they did not meet all eligibility criteria (one patient treated with lenalidomide plus rituximab had not relapsed while on ibrutinib and one patient was not treated with lenalidomide); these two patients are not included in the overall enrolled set of 58 patients.

Patients had a median age of 71 years (range, 50–89), and 71% were aged ≥ 65 years (Table 1). Forty-eight percent of patients had an Eastern Cooperative Oncology Group performance status of 0–1, 29% had high tumor burden, and 14% had bulky disease (≥ 7 cm). The Mantle Cell International Prognostic Index (MIPI) score could not be derived for most patients due to a lack of the required data to complete appropriate calculations for 30 patients (i.e., 52% missing data for MIPI; Ki-67 data were not collected).

**Table 1:** Patient characteristics at study entry

Characteristic	L (*n* = 13)		L + R (*n* = 11)		L + other (*n* = 34)		Overall (*N* = 58)	
	No.	%	No.	%	No.	%	No.	%
Median age, years (range)	67 (54–83)		70 (58–84)		71 (50–89)		71 (50–89)	
≥ 65	6	46	9	82	26	76	41	71
Sex								
Male	11	85	8	73	25	74	44	76
Female	2	15	3	27	9	26	14	24
ECOG PS								
0–1	7	54	5	45	16	47	28	48
2–4	3	23	1	9	4	12	8	14
Missing	3	23	5	45	14	41	22	38
Tumor burden^a^								
High	4	31	1	9	12	35	17	29
Low	1	8	5	45	13	38	19	33
Missing	8	62	5	45	9	26	22	38
Bulky disease^b^								
Yes	2	15	0	0	6	18	8	14
No	2	15	6	55	17	50	25	43
Missing	9	69	5	45	11	32	25	43
Time from diagnosis to first lenalidomide dose, months								
Median	58		47		46		49	
Range	15–144		6–105		4–214		4–214	
Time from end of last prior antilymphoma therapy to first dose of lenalidomide, weeks								
Median	0.7		0.3		0.7		0.7	
Range	0.1–3.5		0.1–21.7		0.1–12.6		0.1–21.7	

*ECOG PS* Eastern Cooperative Oncology Group performance status, *L* lenalidomide, *L + R* lenalidomide plus rituximab

^a^High tumor burden is defined as at least one lesion ≥ 5 cm in diameter or three lesions ≥ 3 cm in diameter [22]

^b^Bulky disease is defined as at least one lesion ≥ 7 cm in the longest diameter^22^

Patients had received a median of four prior lines of systemic anti-lymphoma therapy (range, 1–13), 88% had three or more prior therapies, and 79% had received ibrutinib as monotherapy (Table 2). Most patients (60%) had lenalidomide-containing therapy as their next line of therapy, and 40% patients had ≥ 1 line(s) of other therapy preceding the lenalidomide regimen. Median duration of ibrutinib treatment was 4.3 months (range, 0.5–47.6). Eighty-eight percent of patients discontinued ibrutinib treatment for one or more reason, due to relapse/PD (*n* = 27) and/or refractoriness (*n* = 25), six patients discontinued due to toxicity, and one patient completed ibrutinib as planned but had relapsed/PD at the end of ibrutinib treatment. Besides ibrutinib, the most common previous systemic therapies were rituximab (97%), cyclophosphamide (84%), glucocorticoids (78%), vincristine (78%), doxorubicin (72%), bendamustine (57%), and cytarabine (52%) (Additional file 1: Table S3; note that multiple treatment names could be used to collect this information). The median time from last dose of ibrutinib to first dose of lenalidomide was 1.3 weeks (range, 0.1–21.7) (Table 3).

**Table 2:** Treatment history of enrolled patients

	L (*n* = 13)		L + R (*n* = 11)		L + other (*n* = 34)		Overall (*N* = 58)	
	No.	%	No.	%	No.	%	No.	%
No. of prior antilymphoma treatment regimens								
Median	4		3		4		4	
Range	3–7		2–8		1–13		1–13	
No. of prior antilymphoma therapies								
1	0	0	0	0	1	3	1	2
2	0	0	4	36	2	6	6	10
3	5	38	3	27	10	29	18	31
≥ 4	8	62	4	36	21	62	33	57
Missing	0	0	0	0	0	0	0	0
Type of ibrutinib treatment								
Combination regimen	1	8	1	9	10	29	12	21
Monotherapy	12	92	10	91	24	71	46	79
Ibrutinib status at study inclusion								
Relapse/PD	6	46	2	18	15	44	23	40
Refractory	2	15	8	73	15	44	25	43
Intolerant	3	23	0	0	3	9	6	10
Missing	2	15	1	9	1	3	4	7
Duration of ibrutinib treatment, months								
Median	4.8		3.9		4.3		4.3	
Range	1.2–13.9		2.0–16.6		0.5–47.6		0.5–47.6	
Best response on ibrutinib								
CR	2	15	0	0	6	18	8	14
PR	5	38	2	18	11	32	18	31
SD	0	0	1	9	0	0	1	2
Relapse/PD	5	38	8	73	15	44	28	48
Unknown	1	8	0	0	2	6	3	5
Primary reason for ibrutinib discontinuation								
Lack of efficacy	9	69	11	100	31	91	51	88
Toxicity to ibrutinib	3	23	0	0	2	6	5	9
Toxicity attribution unknown	0	0	0	0	1	3	1	2
Completed ibrutinib treatment	1	8	0	0	0	0	1	2
Time from end of last dose of ibrutinib to first dose of lenalidomide, weeks^a^								
Median	1.4		0.4		1.3		1.3	
Range	0.1–7.4		0.1–21.7		0.1–16.8		0.1–21.7	

*CR* complete response, *L* lenalidomide, *L + R* lenalidomide plus rituximab, *PD* progressive disease, *PR* partial response, *SD* stable disease

^a^Time from last dose of ibrutinib to first dose of lenalidomide (weeks) is calculated as (lenalidomide first dose date − end date of ibrutinib + 1)/7

**Table 3:** Efficacy outcomes with lenalidomide in patients with MCL after ibrutinib failure or intolerance

Outcome	L (*n* = 13)		L + R (*n* = 11)		L + other^a^ (*n* = 34)		Overall (*N* = 58)	
	No.	%	No.	%	No.	%	No.	%
Best response by investigator’s assessment								
ORR	2	15	3	27	12	35	17	29
95% CI	2–45%		6–61%		20–54%		18–43%	
CR	0	0	1	9	7	21	8	14
PR	2	15	2	18	5	15	9	15
SD	0	0	1	9	3	9	4	7
Relapse/PD	8	62	3	27	16	47	27	47
Unknown	3	23	2	18	3	9	8	14
Missing	0	0	2	18	0	0	2	3
Duration of response, weeks								
KM median	3		20		NA		20	
95% CI	NA to NA		NA to NA		16.4 to NA		2.9 to NA	

*CI* confidence interval, *CR* complete response, *KM* Kaplan-Meier, *L* lenalidomide, *L + R* lenalidomide plus rituximab, *MCL* mantle cell lymphoma, *NA* not applicable, *PD* progressive disease, *PR* partial response, *SD* stable disease

^a^Additional file 1: Table S2 lists the other treatments

### Efficacy

Among the 58 patients, the median duration of treatment was 8.4 weeks for single-agent lenalidomide and 7.4 weeks for lenalidomide-containing combination therapy (Table 4). Eight patients achieved a CR and nine achieved a PR with lenalidomide-based therapy, for an ORR of 29% (95% CI, 18–43%; Table 3), which exceeded the predefined lower boundary of the 95% confidence threshold of 10% ORR. Seven of the eight patients with CR had CT ± PET/CT assessments. Two of the 13 patients (15%) who had single-agent lenalidomide (fourth line of therapy for both) reported a best response of relapse/PD to ibrutinib; 3/13 (23%) patients on single-agent lenalidomide had unknown response status with 8/13 (62%) reporting relapse/PD.

**Table 4:** Lenalidomide treatment exposure (safety population)

	L (*n* = 13)	L + R (*n* = 11)	L + other (*n* = 34)	Overall (*N* = 58)
Lenalidomide treatment duration, weeks				
Median	8.4	14.0	7.0	8.4
Range	0.4 to 30.0	0.9 to 37.9	1.1 to 77.9	0.4 to 77.9
Number of lenalidomide cycles				
Median	2.0	2.0	1.0	2.0
Range	1.0 to 7.0	1.0 to 9.0	0.0 to 11.0	0.0 to 11.0
Duration of other therapy combined with lenalidomide, weeks				
Median	NA	8.3	7.2	7.4
Range	NA	0.1 to 35.9	0.7 to 77.7	0.1 to 77.7

*L* lenalidomide, *L + R* lenalidomide plus rituximab, *NA* not applicable

The median DOR for responders was 20 weeks (95% CI, 2.9 to not reached); of the 17 responders, 14 (82%; 7 CR and 7 PR) were censored from the DOR analysis due to lack of follow-up data on PD or death. At the last available assessment of the 14 censored patients, three were ongoing, three had completed lenalidomide treatment as planned, and eight patients discontinued lenalidomide treatment early (withdrew consent [*n* = 1], patient decision [*n* = 1], enrolled in a clinical trial for oral treatment [*n* = 1], started other lines of treatment [*n* = 3; because of lung cancer, physician’s decision, or bone marrow transplant], and toxicity [*n* = 2]). One of the censored patients who had a first response of PR and best response of CR had the last censored DOR at 25 weeks before stopping therapy. For the three uncensored patients, two had a best response of PR and one had CR, with an estimated DOR of 2.9, 19.7, and 16.4 weeks, respectively. Univariate analysis showed a median DOR of 16 weeks (95% CI, 2.9–19.7) in the three uncensored patients (14 patient responders were censored; total of 17 responders).

### Response by subgroup analysis

**Fig. 1:**
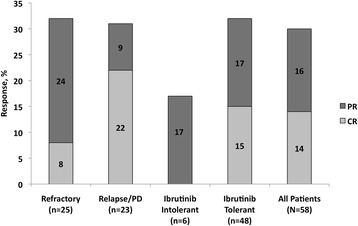
Best evaluable response to lenalidomide by subgroup. Subgroups include those of refractory versus relapsed/progressive disease, intolerant versus tolerant to ibrutinib, and all patients. CR complete response, PD progressive disease, PR partial response. Response data were missing or unknown for 3 refractory, 5 relapse/PD, 0 ibrutinib intolerant, 8 ibrutinib tolerant, and 10 patients overall

Patients with MCL refractory to ibrutinib versus those who relapsed/progressed on or following ibrutinib had similar ORRs of 32 versus 30%, respectively (Fig. 1); however, the CR rates were not similar (8 versus 22%). The median DOR was 20 weeks (CI 95%, 2.9–20) for the ibrutinib-refractory group and not available for the relapsed/PD group. There was one PR (17%) among the six patients who were ibrutinib-intolerant; all six patients were treated with lenalidomide within 6 months of stopping ibrutinib therapy. Of the 48 patients who tolerated ibrutinib therapy, seven had CRs and eight had PRs, a 31% ORR, and the median DOR was 20 weeks.

### Safety

Overall, patients received a median of two cycles (range, 0–11) of lenalidomide-based treatment. Most patients received lenalidomide 10–25 mg/day on days 1–21 of each 28-day cycle. As of the cutoff date of November 1, 2016, 54 patients had discontinued lenalidomide-based therapy and four patients continue to receive lenalidomide (three censored for efficacy analyses), one in combination with weekly bortezomib/dexamethasone/rituximab, two in combination with weekly rituximab, and one in combination with weekly obinutuzumab. The primary reasons for lenalidomide treatment discontinuation were lack of efficacy (*n* = 27); toxicity (*n* = 10); other reasons (*n* = 9), such as initiation of another therapy (e.g., based on physician or patient choice) or trial (also an oral therapy), undergoing stem cell transplantation, or primary clinician/patient decision to stop therapy; completion of lenalidomide treatment (*n* = 5); and missing data (*n* = 3).

Of the 58 patients analyzed for safety, 48 (83%) had one or more TEAE during lenalidomide treatment. Twenty (34%) patients had at least one serious TEAE (lenalidomide alone 23%; lenalidomide + rituximab 36%; lenalidomide + others 38%). The most frequently reported serious TEAEs of any grade were febrile neutropenia (*n* = 4; 7%), hypotension (7%), deep vein thrombosis (DVT) (*n* = 3; 5%), pneumonia (5%), pancytopenia (5%), fall (5%), acute kidney injury (5%), dyspnea (*n* = 2; 3%), sepsis (3%), and respiratory failure (3%). Overall, nine (16%) patients had at least one TEAE leading to dose discontinuation (lenalidomide alone 8%; lenalidomide + rituximab 18%; lenalidomide + others 18%). These TEAEs included pancytopenia, thrombocytopenia, and rash, each experienced by two patients (3%), and anemia, febrile neutropenia, neutropenia, sepsis, fall, squamous cell lung carcinoma, dyspnea, pleural effusion, and orthostatic hypotension, each experienced by one patient (2%). The most common all-grade TEAEs were fatigue, cough, dizziness, dyspnea, nausea, peripheral edema, anemia, rash, thrombocytopenia, and neutropenia (Table 5).

**Table 5:** Documented treatment-emergent all-grade adverse events in ≥ 10% of patients (safety population)

	L (*n* = 13)		L + R (*n* = 11)		L + other (*n* = 34)		Overall (*N* = 58)	
	No.	%	No.	%	No.	%	No.	%
Hematologic								
Anemia	2	15	3	27	5	15	10	17
Thrombocytopenia	1	8	1	9	7	21	9	16
Neutropenia	1	8	1	9	6	18	8	14
Pancytopenia	1	8	3	27	3	9	7	12
Febrile neutropenia	0	0	0	0	6	18	6	10
Nonhematologic								
Fatigue	4	31	4	36	14	41	22	38
Nausea	2	15	2	18	7	21	11	19
Dizziness	2	15	2	18	7	21	11	19
Dyspnea	2	15	3	27	6	18	11	19
Peripheral edema	0	0	2	18	9	26	11	19
Rash	2	15	1	9	7	21	10	17
Cough	1	8	3	27	7	21	11	19
Decreased appetite	2	15	0	0	5	15	7	12
Diarrhea	0	0	1	9	7	21	8	14
Headache	3	23	1	9	2	6	6	10
Pyrexia	1	8	0	0	5	15	6	10
Vomiting	0	0	2	18	4	12	6	10
Constipation	0	0	0	0	6	18	6	10
Laboratory investigations								
Platelet count decreased	2	15	1	9	3	9	6	10
White blood cell count decreased	1	8	1	9	4	12	6	10

*L* lenalidomide, *L + R* lenalidomide plus rituximab

As of the cutoff date, 28 (48%) patients had died, 12 (21%) during treatment with lenalidomide, and 15 (26%) during follow-up (one unknown). Overall, 20 (34%) patients died from malignant disease (i.e., MCL) or its complications, five from unknown causes (not assessable or insufficient data), one reported another cause of end-stage renal disease, and two due to AEs. Of the two patients who died due to AEs, the first patient included a 68-year-old man in the lenalidomide-alone group who died during treatment (83 days after the first lenalidomide dose). This patient had a PR 2 months after lenalidomide initiation but died due to a pulmonary embolism, suspected to be related to lenalidomide therapy, as well as had incidences of other grade 5 AEs (DVT and cardiac arrest). Although this patient was receiving aspirin, therapy was stopped during study admission. For most patients, it is not known if the patients received antithrombotic treatment, since concomitant treatments were not part of the collected data. The second patient who died due to an AE was a 71-year-old man who received one treatment cycle of lenalidomide in combination with ibrutinib, rituximab, bortezomib, and dexamethasone. This second patient died while on study treatment (25 days after the first dose of lenalidomide) because of progression of MCL (which included acute kidney injury, lactic acidosis, respiratory failure, and hypotension).

## Discussion

This multicenter observational study examined outcomes with lenalidomide treatment in patients with MCL who had relapsed or progressed after or during ibrutinib therapy or were intolerant to ibrutinib. Most patients had received three or more prior lines of treatment and had discontinued ibrutinib due to a lack of efficacy. Most patients (79%) had previously received ibrutinib as a monotherapy. The ORR of 45% and median DOR of 4.3 months were lower than in previous clinical trials of ibrutinib monotherapy for relapsed/refractory MCL; there were also a higher number of prior regimens in the current study [34, 35]. These factors suggest a higher-risk cohort and a potential negative impact on response to subsequent therapy, including lenalidomide. Nonetheless, lenalidomide-based treatment demonstrated meaningful clinical activity in this difficult-to-treat patient population, as demonstrated by a 29% ORR and 14% CR, with a 20-week (95% CI, 2.9 to not available) median DOR. For the DOR analysis, it should be noted that because 82% of responders were censored, the data should be interpreted with caution. With no new safety signals identified, the safety profile in these patients matched the well-established safety shown in multiple studies of lenalidomide monotherapy [22–28].

Prior studies have shown that lenalidomide treatment had significant clinical activity in relapsed/refractory MCL. The MCL-001 EMERGE study reported a 28% ORR (including 8% CR/CR unconfirmed) and 16.6-month DOR with lenalidomide monotherapy in 134 patients with relapsed/refractory MCL after bortezomib treatment. Patients from MCL-001 had received a median of four prior treatment regimens, and 88% had been treated with at least three prior systemic antilymphoma therapies [26]. A UK study reported a 31% ORR, 8% CR, and 22.2-month median DOR with single-agent lenalidomide (6 cycles at 25 mg/day followed by 15 mg/day lower maintenance dose) in 26 patients with relapsed/refractory MCL who had received a median of three prior systemic therapies [25]. The lower DOR of < 5 months in the current study could be a result of ibrutinib resistance. In the randomized MCL-002 (SPRINT) study of 254 patients with relapsed/refractory MCL, the lenalidomide monotherapy group showed higher ORR (40 versus 11%; *P* < 0.001) compared with investigator’s choice (monotherapy with chlorambucil, cytarabine, gemcitabine, fludarabine, or rituximab), respectively [28]. Median DOR was 16.1 months for lenalidomide and 10.4 months for the investigator’s choice group. Lenalidomide in combination with rituximab (R^2^) has also shown activity in relapsed/refractory MCL. In a phase I/II dose-finding study, R^2^ was well tolerated in MCL, and among 44 patients in phase II, ORR was 57% (CR 36%) and DOR was 18.9 months [30]. A phase II study of iNHL or MCL showed lenalidomide monotherapy followed by R^2^ overcame rituximab resistance [29]. In the 14 patients with MCL, ORR after lenalidomide monotherapy and R^2^ was 55% for each; DOR to R^2^ was 22.1 months. Since responses to lenalidomide in the post-ibrutinib setting are not durable, early referral for allogeneic hematopoietic stem cell transplantation (allo-HCT) should be strongly considered for responding MCL patients without advanced comorbidities [36–38].

There are several limitations to the study, including the retrospective nature of chart review and limited follow-up, which contribute to censoring patients for time-to-event statistics such as DOR. The prevalence of AEs may also be underestimated due to possible under-reporting or other uncontrolled factors such as pre-existing events. Safety summary tables were generated with the expectation of missing data (e.g., grade, treatment-relatedness, seriousness) that might limit the safety analysis. Because of the heterogeneity of regimens combined with lenalidomide, it is difficult to confidently discern the amount of response due to lenalidomide versus the other therapies used in combination, apart from two responses to lenalidomide monotherapy. The two responders to lenalidomide monotherapy represented only 12% of the 17 patients who responded on lenalidomide-containing therapy, further complicating delineation of the effects of lenalidomide with or without other therapies. It would also be beneficial to deduce which patients were previously refractory to rituximab.

As ibrutinib is being used more frequently for patients with MCL, the opportunity now arises to assess the role of other therapies following ibrutinib. Because multiple studies have shown that MCL patients with ibrutinib failure demonstrate poor outcomes with subsequent therapy [16, 17]; it is critical to identify therapies that may provide activity in these patients. Multiple second-generation BTK inhibitors are being investigated to evaluate possible improvements in target specificity, potency, and tolerability through this pathway [39, 40].

## Conclusion

Results from this observational study indicate that lenalidomide-based therapy has clinically significant activity as a monotherapy and in combination regimens to treat heavily pretreated patients with refractory or relapsed MCL after ibrutinib therapy or who cannot tolerate ibrutinib, and thus, lenalidomide addresses an unmet medical need and widens the therapeutic options in a difficult-to-treat patient population.

## Additional files

Additional file 1: Table S1. Number of patients per study site. **Table S2.** Lenalidomide combination treatments for L + other group (*n* = 34). Table S3 Prior systemic anti-lymphoma therapies (≥ 10% of patients; *N* = 58)*. (DOCX 42 kb)
