# Supplementary material for: Observational study of lenalidomide in patients with mantle cell lymphoma who relapsed/progressed after or were refractory/intolerant to ibrutinib (MCL-004)
Source: J Hematol Oncol. 2017 Nov 2;10:171. doi: 10.1186/s13045-017-0537-5 (PMC5668956; doi:10.1186/s13045-017-0537-5)
Supplement: Supplementary file 1 — Number of patients per study site. Table S2. Lenalidomide combination treatments for L + other group (n = 34). Table S3 Prior systemic anti-lymphoma therapies (≥ 10% of patients; N = 58)*. (DOCX 42 kb) [file 13045_2017_537_MOESM1_ESM.docx]

**Additional Supplemental Files**

**Supplemental Table 1** Number of patients per study site

|  | L (n=13) | | L+R (n=11) | | L+Other (n=34) | | Overall (N=58) | |
| --- | --- | --- | --- | --- | --- | --- | --- | --- |
|  | **No** | **%** | **No** | **%** | **No** | **%** | **No** | **%** |
| Univ. of Texas MDACC | 0 | 0 | 3 | 27 | 16 | 47 | 19 | 33 |
| Weill Cornell Medical College | 3 | 23 | 3 | 27 | 7 | 21 | 13 | 22 |
| Univ. of Michigan Comprehensive Cancer Center | 3 | 23 | 1 | 9 | 1 | 3 | 5 | 9 |
| Sylvester Comprehensive Cancer Center | 2 | 15 | 2 | 18 | 1 | 3 | 5 | 9 |
| Froedtert and The Medical College of Wisconsin | 1 | 8 | 0 | 0 | 3 | 9 | 4 | 7 |
| Derriford Hospital | 3 | 23 | 0 | 0 | 1 | 3 | 4 | 7 |
| Hackensack Univ. Medical Center | 0 | 0 | 0 | 0 | 3 | 9 | 3 | 5 |
| Univ. of Pennsylvania | 0 | 0 | 0 | 0 | 2 | 6 | 2 | 3 |
| Mayo Clinic Scottsdale | 1 | 8 | 0 | 0 | 0 | 0 | 1 | 2 |
| Levine Cancer Center | 0 | 0 | 1 | 9 | 0 | 0 | 1 | 2 |
| Non Engaged-First Health of the Carolinas | 0 | 0 | 1 | 9 | 0 | 0 | 1 | 2 |

L: lenalidomide; L+R: lenalidomide plus rituximab; MDACC: MD Anderson Cancer Center; Univ.: University.

**Supplemental Table 2** Lenalidomide combination treatments for L+Other group (n=34)

| Lenalidomide Plus: | No |
| --- | --- |
| Bortezomib/dexamethasone/rituximab | 6 |
| Bortezomib/dexamethasone/ibrutinib/rituximab | 3 |
| Carfilzomib/dexamethasone/rituximab | 3 |
| Bortezomib/rituximab | 2 |
| Dexamethasone/bortezomib | 2 |
| Dexamethasone/ibrutinib/obinutuzumab | 2 |
| Dexamethasone/rituximab | 2 |
| Ibrutinib | 2 |
| Rituximab/vincristine | 2 |
| Bendamustine | 1 |
| Bendamustine/rituximab/vincristine | 1 |
| Bortezomib | 1 |
| Bortezomib/dexamethasone/ibrutinib | 1 |
| Cytarabine | 1 |
| Dexamethasone/cyclophosphamide | 1 |
| Dexamethasone/everolimus/ibrutinib | 1 |
| Dexamethasone/obinutuzumab | 1 |
| Obinutuzumab | 1 |
| Prednisone/rituximab | 1 |

**Supplemental Table 3** Prior systemic anti-lymphoma therapies (≥10% of patients; N=58)*

| Description | No (%) |
| --- | --- |
| **Protein kinase inhibitors**  Ibrutinib  Palbociclib | **58 (100)**  58 (100) 8 (14) |
| **Monoclonal antibodies**  Rituximab | **56 (97)** 56 (97) |
| **Alkylating agents**  Cyclophosphamide  Bendamustine  Ifosfamide | **56 (97)**  49 (84) 33 (57) 7 (12) |
| **Glucocorticoids** Dexamethasone  Prednisone  Prednisolone | **45 (78)** 29 (50) 17 (29) 7 (12) |
| **Vinca alkaloids and analogues**  Vincristine/vincristine sulfate | **45 (78)** 45 (78) |
| **Anthracyclines and related substances**  Doxorubicin/doxorubicin hydrochloride | **42 (72)**  42 (72) |
| **Other antineoplastic agents**   Bortezomib | **30 (52)** 29 (50) |
| **Pyrimidine analogues**  Cytarabine | **30 (52)**  30 (52) |
| **Folic acid analogues**  Methotrexate | **24 (41)** 24 (41) |
| **Podophyllotoxin derivatives**  Etoposide | **14 (24)** 14 (24) |
| **Platinum compounds**  Cisplatin | **11 (19)** 8 (14) |
|  |  |

*2 patients total (1 each in the L+R and L+Other group) had received prior lenalidomide therapy.
